# Dynamic hip kinematics during recreational classical ballet and hula dance after total hip arthroplasty: two case reports

**DOI:** 10.1186/s13256-018-1942-2

**Published:** 2019-01-12

**Authors:** Keisuke Komiyama, Satoshi Hamai, Daisuke Hara, Satoru Ikebe, Yifeng Wang, Hirotaka Gondo, Hidehiko Higaki, Yasuharu Nakashima

**Affiliations:** 1 Department of Orthopedic Surgery, Graduate School of Medical Sciences, Kyushu University, 3-1-1 Maidashi, Higashi-ku, Fukuoka, 812-8582 Japan; 2 Department of Life Science, Faculty of Life Science, Kyushu Sangyo University, 2-3-1 Matsugadai, Higashi-ku, Fukuoka, 813-0004 Japan; 3 Department of Creative Engineering, National Institute of Technology, Kitakyushu College, 5-20-1 Shii, Kokuraminami-ku, Kitakyushu, Fukuoka 802-0985 Japan

**Keywords:** Hula, Ballet, Dance, Total hip arthroplasty, Kinematics, Image-matching

## Abstract

**Background:**

The *in vivo* assessment of the three-dimensional kinematics of the hip during dance activities in patients after total hip arthroplasty has not been previously reported. We evaluated the replaced hip kinematics during recreational classical ballet and hula dance using radiographic-based image-matching techniques.

**Case presentation:**

A 58-year-old Japanese woman (patient 1; height, 157 cm; weight, 74.5 kg) and a 73-year-old Japanese woman (patient 2; height, 153 cm; weight, 48 kg) were still doing classical ballet and hula dance, respectively, after primary total hip arthroplasty. For ballet, there were gradual three-dimensional hip movements with 48° flexion, 36° abduction, and 49° external rotation in développé and 34° flexion, 29° abduction, and 43° external rotation in plié. For hula, there were small three-dimensional hip movements with 31° flexion, 15° adduction, and 11° external rotation in kao and 17° flexion, 11° adduction, and 11° external rotation in kaholo. No liner-to-neck contact was found in any dance activities.

**Conclusion:**

Both classical ballet and hula dance produced complex ranges of hip movements and activity-dependent kinematics. These kinematic data could be useful for recommending each patient with total hip arthroplasty to continue recreational dance activities.

## Background

Total hip arthroplasty (THA) provides pain relief, deformity correction and restored function, and promotes good long-term outcomes [1–3]. The clinical success of THA allows some patients to participate in sports activities and expectations of returning to sports activities have become more important to patients than ever before [4–6]. Participation in physical activities has positive effects on patients, such as improved bone quality, muscle strength, flexibility, and implant fixation [7–9].

Healy *et al.* reported that a survey conducted in 2005 revealed that members of The Hip Society allowed square dancing after THA [4]. Ollivier *et al*. reported that 93 patients out of 571 patients (16.3%) enjoyed dancing activities after THA [5]. However, concerns exist about whether dance can be performed in a safe manner after THA. To the best of our knowledge, no previous report is available that studies the *in vivo* three-dimensional kinematics of dance after THA. Image-matching techniques have provided reliable information on three-dimensional hip joint range of motion (ROM) during daily and sports activities [10–15]. Based on the kinematic data, patients after THA could be instructed regarding risks associated with specific postures [12].

The purpose of this study was to measure dynamic hip kinematics during classical ballet and Hawaiian dance after THA using image-matching techniques. The following question was addressed: What values of flexion/extension, adduction/abduction, and axial rotation are produced at the hip joint?

## Case presentation

A 58-year-old Japanese woman (patient 1; height, 157 cm; weight, 74.5 kg; body mass index, 30.2 kg/m^2^) and a 73-year-old Japanese woman (patient 2; height, 153 cm; weight, 48 kg; body mass index, 20.5 kg/m^2^) were still doing classical ballet and hula dance, respectively, after primary THA for osteoarthritis (OA) due to developmental dysplasia of the hip. They received medication and rehabilitation prior to surgery for 2 and 10 years, respectively. Patient 1 could not do classical ballet before surgery, and returned to doing classical ballet recreationally with satisfaction after surgery. Patient 1’s preoperative Oxford Hip Score (OHS) [16, 17] and University of California-Los Angeles (UCLA) activity scale score [17, 18] were 4 and 1, respectively. Patient 2 did hula dance with difficulty due to right coxalgia, and enjoyed hula dancing after surgery. Patient 2’s preoperative OHS and UCLA score were 4 and 2, respectively. The occupation of both patients was homemaker. The OHS, the UCLA score, and the Harris Hip Score (HHS) [19] in patient 1 were 48, 8, and 100, respectively, at 4 years of follow-up after surgery. The OHS, the UCLA score, and the HHS in patient 2 were 48, 5, and 80, respectively, at 6 years of follow-up after surgery. The OHS and UCLA score are validated, reliable, and self-reported metric assessments for patients with hip OA [16–18]. The OHS assesses the pain and function of the hip during daily activities, while the UCLA score measures physical activity levels. Both patients provided written consent for this institutional review board-approved study and were willing to participate and enroll in the study.

A cementless hemispherical press fit cup, straight metaphyseal fit stem, alumina ceramic femoral heads (patient 1, 32 mm; patient 2, 26 mm), and highly cross-linked ultra-high molecular weight polyethylene liner with a 15° elevated rim (AMS® and PerFix HA, Aeonian; Kyocera Medical, Osaka, Japan) were used [2, 3, 12]. All operations were performed using combined anteversion technique via a posterolateral approach [20, 21].

**Fig. 1:**
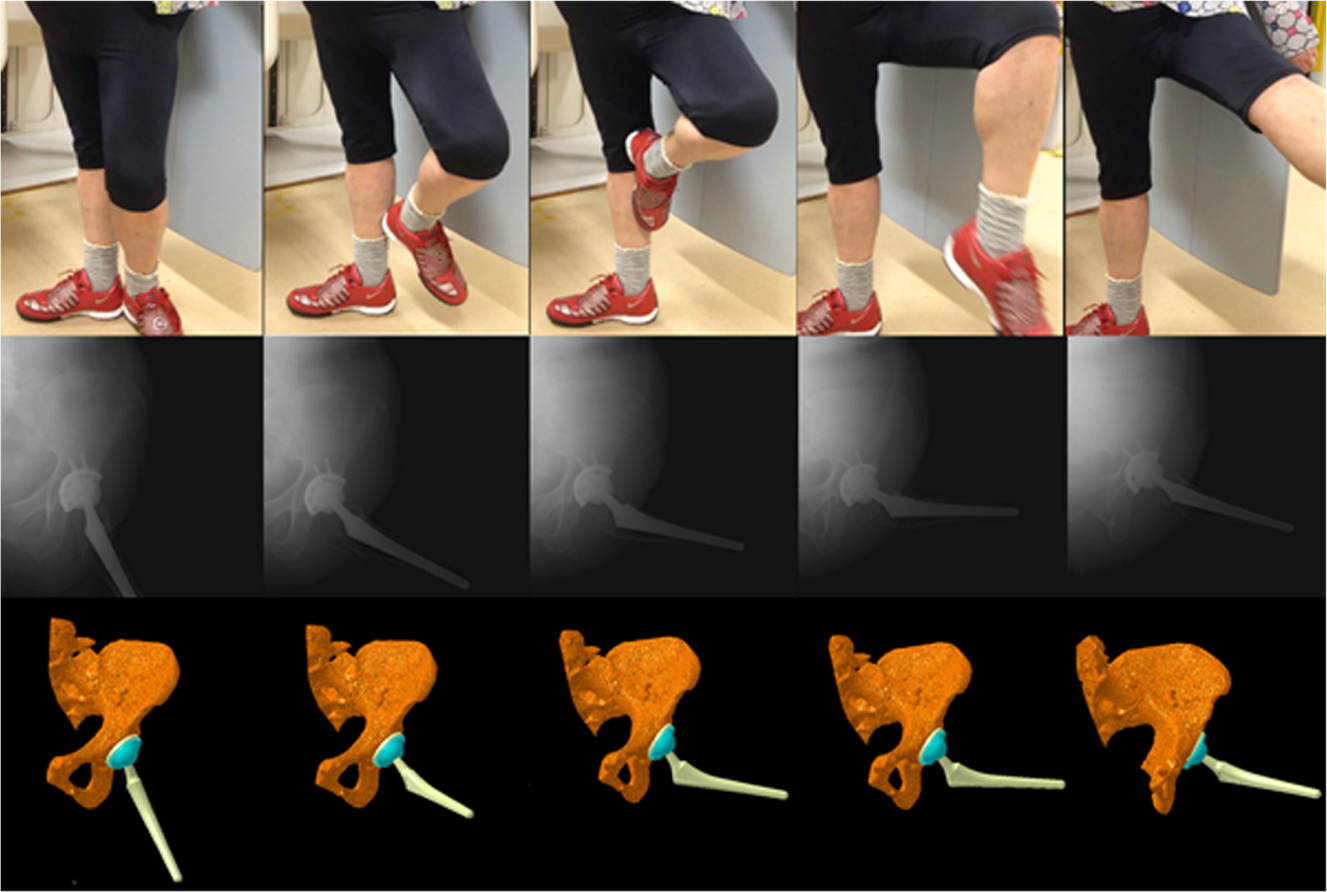
The hip motions during the ballet movement développé were captured as continuous X-ray images using a flat panel X-ray detector (*middle stand*) to reconstruct three-dimensional images of the replaced hip joint (*lower stand*) using image-matching techniques

**Fig. 2:**
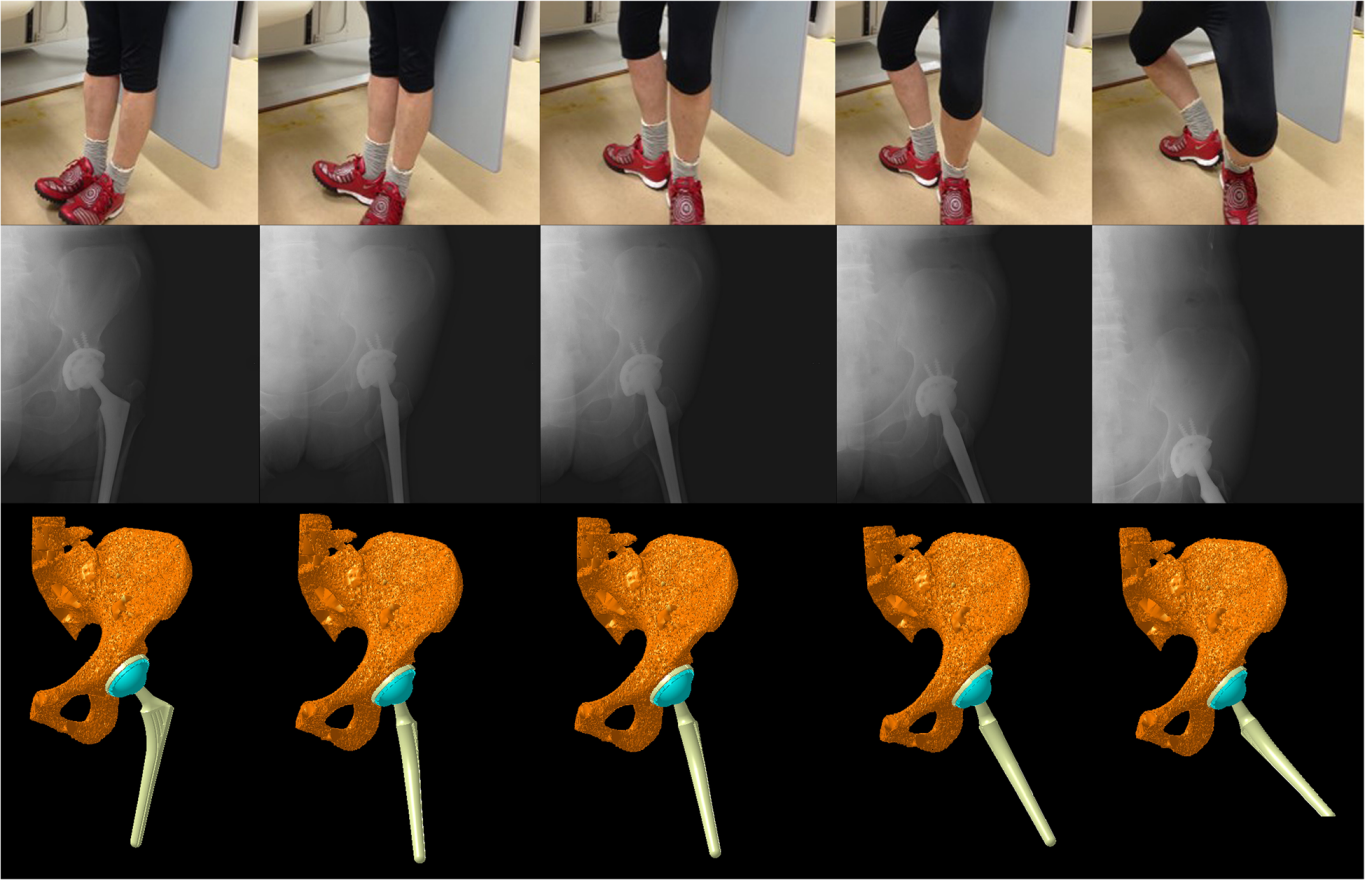
The hip motions during the ballet movement plié were captured as continuous X-ray images using a flat panel X-ray detector (*middle stand*) to reconstruct three-dimensional images of the replaced hip joint (*lower stand*) using image-matching techniques

**Fig. 3:**
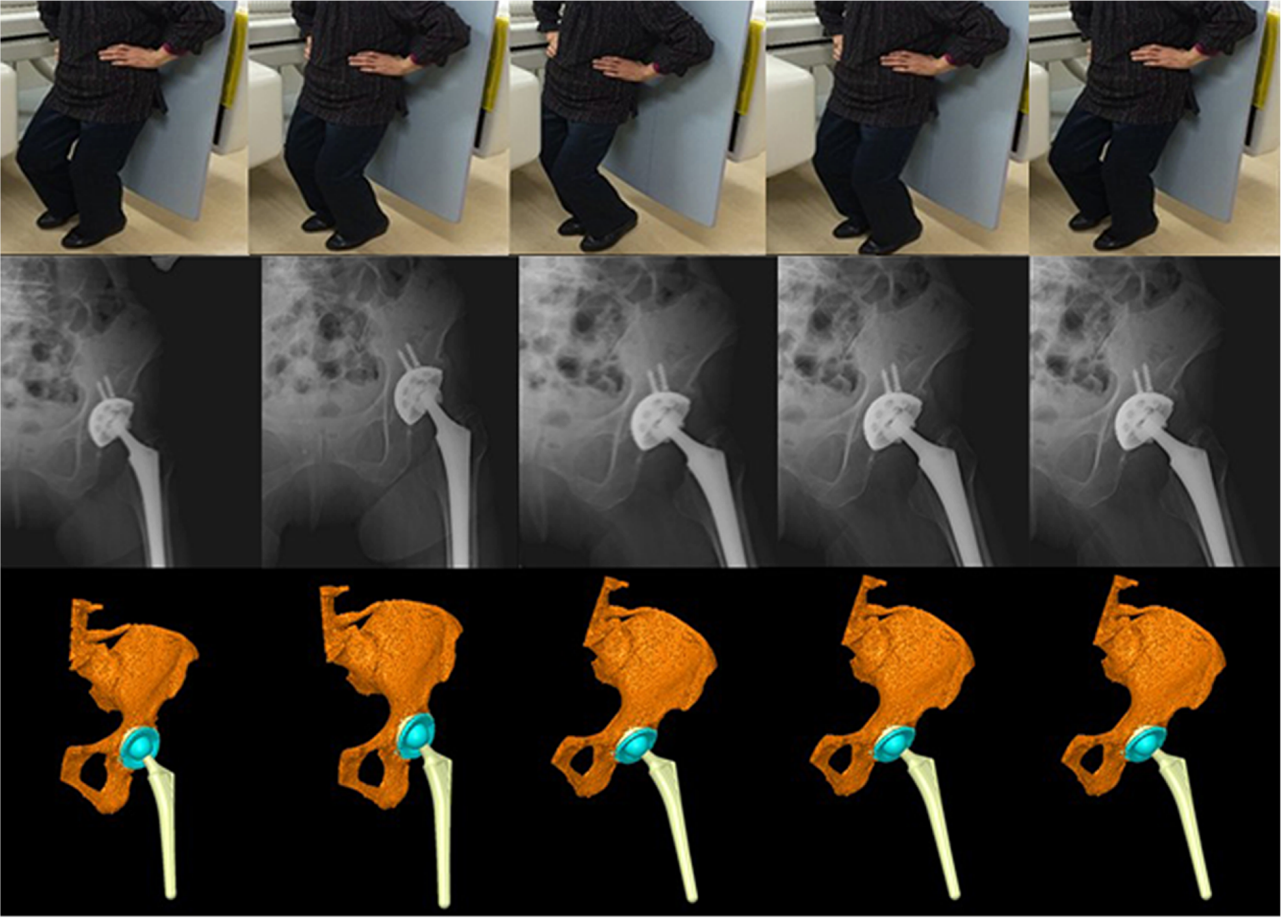
The hip motions during the hula dance movement kao were captured as continuous X-ray images using a flat panel X-ray detector (*middle stand*) to reconstruct three-dimensional images of the replaced hip joint (*lower stand*) using image-matching techniques

**Fig. 4:**
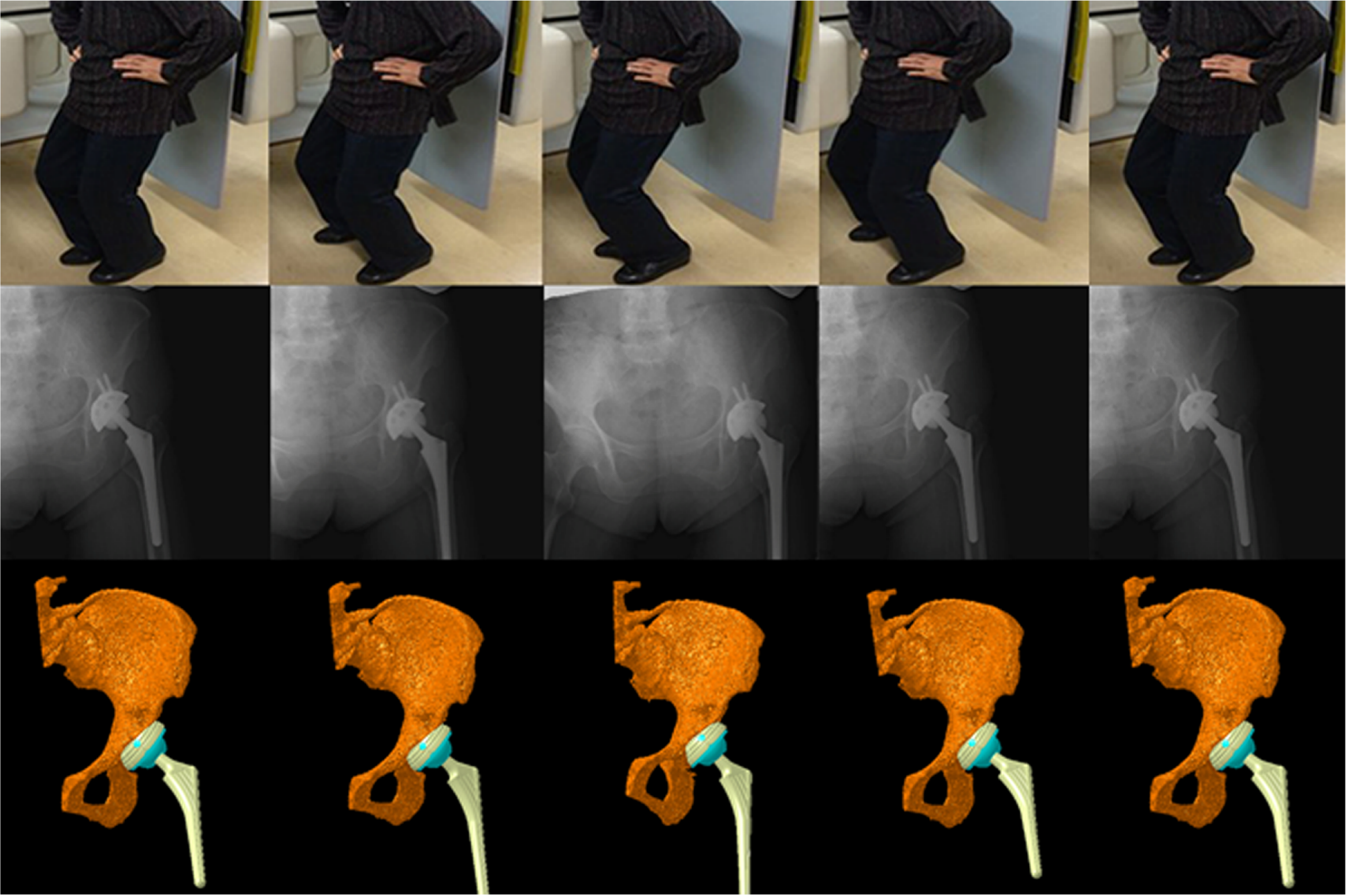
The hip motions during the hula dance movement kaholo were captured as continuous X-ray images using a flat panel X-ray detector (*middle stand*) to reconstruct three-dimensional images of the replaced hip joint (*lower stand*) using image-matching techniques

The three-dimensional positions and orientations of the pelvis, acetabular cup, femur, and femoral stem during dance were determined using image-matching techniques [10]. The patients performed dance under continuous radiographic surveillance using a flat panel X-ray detector (Ultimax-I, Toshiba, Tochigi, Japan): image area, 420 mm × 420 mm; resolution, 0.274 mm × 0.274 mm/pixel; and frame rate, 3.5 frames/second (Figs. 1, 2, 3, and 4). Each patient routinely underwent computed tomography (CT; Aquilion, Toshiba, Tochigi, Japan) with a 512 × 512 image matrix, 0.35 × 0.35 pixel dimensions, and 1-mm slice thickness from the superior edge of the pelvis to just below the knee joint line. Anatomical coordinate systems of the pelvis and femur were embedded in each bone model derived from CT data according to our previous study [12]. Computer simulation was performed to generate virtual digitally reconstructed radiographs (DRRs), in which the light source and projected plane parameters were set to be identical to the actual radiographic imaging conditions. Each model silhouette was matched with the actual silhouette by translating and rotating the three-dimensional model to minimize the number of unmatched pixels between the silhouettes. The orientation of the femur relative to the pelvis: hip movements, was determined using the Cardan/Euler angle system in *x*-*y*-*z* order (flexion/extension, adduction/abduction, internal/external rotation). Contact between the acetabular liner and the stem neck (liner-to-neck contact) was also evaluated using a computer-aided design (CAD) software program (CATIA V5; Dassault Systèmes). The maximum errors associated with tracking the position of the femur/stem relative to the pelvis/acetabular cup were 0.36/0.43 mm, 0.37/0.48 mm, and 0.48°/0.52°, respectively, for in-plane translation, out-of-plane translation, and rotation, respectively [12].

The orientations of the acetabular cup and stem were measured using postoperative CT data. Cup inclination was measured as the angle of abduction using the inter-tear-drop line as the baseline (radiographic inclination). Cup anteversion was measured as the angle of anteversion in the sagittal plane (operative anteversion). Femoral anteversion was measured as the angle of anteversion between the prosthetic femoral neck and transe-epicondylar axis (TEA). The cup inclination, cup anteversion, and stem anteversion in patients 1 and 2 were: 40.1°, 41.0°; 14.4°, 25.9°; and 34.8°, 21.8°, respectively.

### Classical ballet

**Fig. 5:**
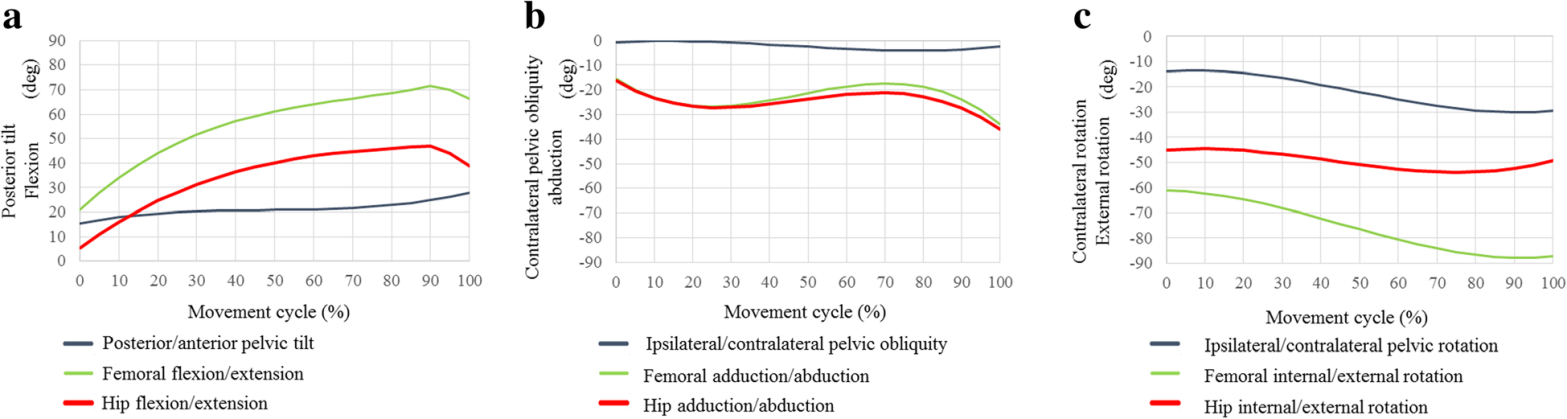
Posterior/anterior pelvic tilt (posterior +, anterior −), and femoral and hip flexion/extension angles (flexion +, extension −) during développé (**a**). Ipsilateral/contralateral pelvic obliquity (ipsilateral +, contralateral −), and femoral and hip adduction/abduction angles (adduction +, abduction −) during développé (**b**). Ipsilateral/contralateral pelvic rotation (ipsilateral +, contralateral −), and femoral and hip internal/external rotation angles (internal +, external −) during développé (**c**)

**Fig. 6:**
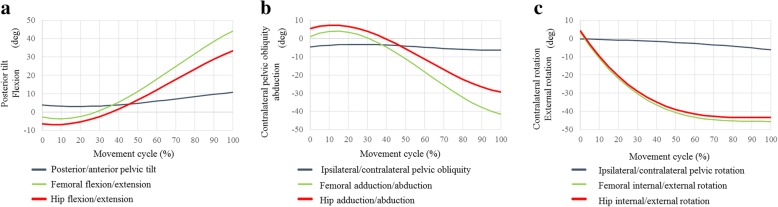
Posterior/anterior pelvic tilt (posterior +, anterior −), and femoral and hip flexion/extension angles (flexion +, extension −) during plié (**a**). Ipsilateral/contralateral pelvic obliquity (ipsilateral +, contralateral −), and femoral and hip adduction/abduction angles (adduction +, abduction −) during plié (**b**). Ipsilateral/contralateral pelvic rotation (ipsilateral +, contralateral −), and femoral and hip internal/external rotation angles (internal +, external −) during plié (**c**)

For the ballet movements of développé (Fig. 1) and plié (Fig. 2), there were gradual three-dimensional hip movements (Figs. 5 and 6). Développé produced 75.3° of maximum femoral flexion with 27.8° of posterior pelvic tilt (Fig. 5). Hip flexion peaked on the way of movement with 47.5° of maximum flexion. The maximum hip abduction was 36.1° with 49.3° of hip external rotation. Plié produced 41.6° of maximum femoral flexion with 10.7° of posterior pelvic tilt in the sagittal plane (Fig. 6). Hip flexion peaked on the way of movement with 33.5° of maximum flexion. The maximum hip abduction was 29.4° with 43.3° of hip external rotation. No liner-to-neck contact was found in either développé or plié.

### Hula dance

**Fig. 7:**
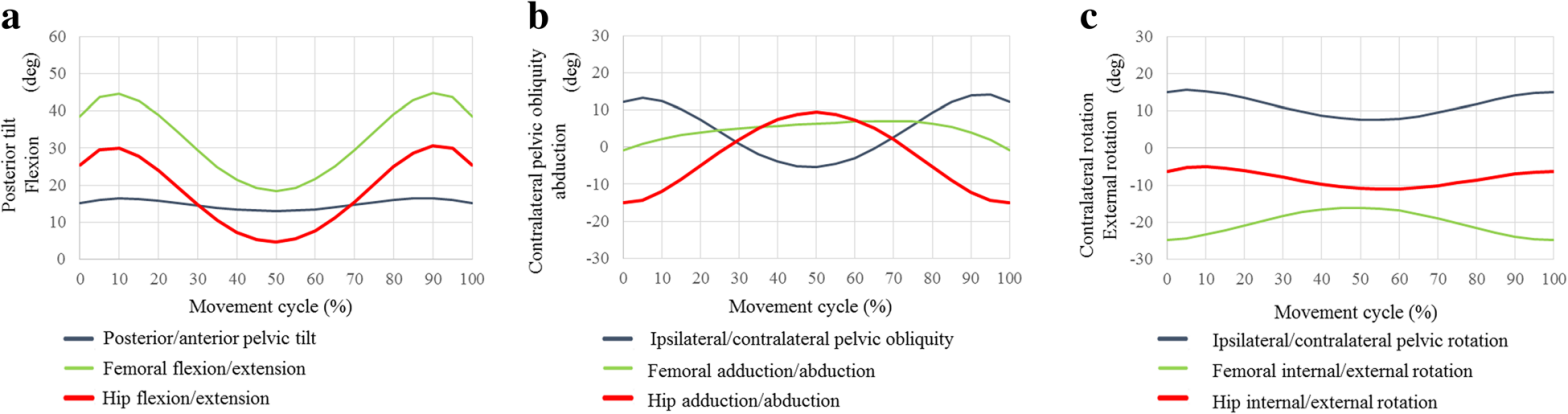
Posterior/anterior pelvic tilt (posterior +, anterior −), and femoral and hip flexion/extension angles (flexion +, extension −) during kao (**a**). Ipsilateral/contralateral pelvic obliquity (ipsilateral +, contralateral −), and femoral and hip adduction/abduction angles (adduction +, abduction −) during kao (**b**). Ipsilateral/contralateral pelvic rotation (ipsilateral +, contralateral −), and femoral and hip internal/external rotation angles (internal +, external −) during kao (**c**)

**Fig. 8:**
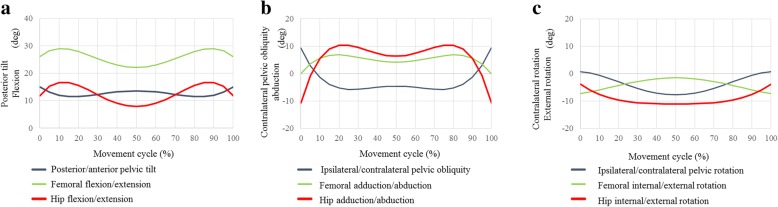
Posterior/anterior pelvic tilt (posterior +, anterior −), and femoral and hip flexion/extension angles (flexion +, extension −) during kaholo (**a**). Ipsilateral/contralateral pelvic obliquity (ipsilateral +, contralateral −), and femoral and hip adduction/abduction angles (adduction +, abduction −) during kaholo (**b**). Ipsilateral/contralateral pelvic rotation (ipsilateral +, contralateral −), and femoral and hip internal/external rotation angles (internal +, external −) during kaholo (**c**)

In the hula dance movement called kao (Fig. 3), hip flexion/extension ranged from 4.6° of flexion to 30.6° of flexion with 15.1° of maximum hip abduction and 11.1° of maximum hip external rotation (Fig. 7). We observed 13.3° of total amount of ipsilateral pelvic obliquity with 16.0° and 15.6° of total amount of posterior pelvic tilt and contralateral pelvic rotation, respectively. In the kaholo (Fig. 4), hip flexion/extension ranged from 7.9° of flexion to 16.7° of flexion with 11° of maximum hip abduction and 10.7° of maximum hip external rotation (Fig. 8). We observed 9.3° of total amount of ipsilateral pelvic obliquity with 15.1° and 0.8° of total amount of posterior pelvic tilt and contralateral pelvic rotation, respectively. No liner-to-neck contact was found in either kao or kaholo.

## Discussion

To the best of our knowledge, this is the first *in vivo* study to examine the dynamic kinematics of the hip joint during dance activities in patients after THA. In this analysis, classical ballet and hula dance produced complex ranges of hip movements and activity-dependent kinematics. For classical ballet, développé/plié produced approximately 48°/34° of maximum hip flexion with 36°/29° of maximum abduction and 49°/43° of maximum external rotation. For hula dance, kao/kaholo produced approximately 31°/17° of maximum flexion with 15°/11° of maximum abduction and 11°/11° of maximum external rotation. No liner-to-neck contact was found in any dance activities.

More than ever before, patients have high expectations in terms of functional outcomes and returning to sports after THA [4]. The present two cases could return to recreational classical ballet and Hula dance with satisfaction after THA. Although there is little information on the kinematic patterns of replaced hip joints during dance activities, these kinematic data should be beneficial for advising patients according to the type of physical activity. Motion capture systems with reflective markers have been widely used for *in vivo* joint kinematics even during classical ballet [22–24]. Quanbeck *et al.* [22] reported that bilateral hip external rotation during turnout in classical ballet was 49°. Hopper *et al*. [23] examined hip adduction angle, thigh tilt angle, and knee–hip distance during five ballet movements: rise, relevé, ballonné en place, ballonné traveling, and sissonne. However, external markers attached to the skin might be affected by soft tissue artefacts, producing substantial errors [25–27]. To the best of our knowledge, our case report provides the first kinematic analysis of dance activity in patients after THA using image-matching techniques.

Maximum hip flexion of approximately 48° during développé that we recorded in this study is less than peak hip flexion values of 81° and 102° previously reported for chair-rising and squatting in healthy hips [10]. Due to the posterior pelvic tilt, hip flexion/extension showed a lower angle relative to the femoral flexion/extension, which is consistent with previous kinematic studies [10]. Compared to the développé, smaller hip flexion/extension, adduction/abduction, and internal/external rotation were found during plié. No excessive hip movement or liner-to-neck contact was found during either développé or plié. Specific posture might include potential risks of prosthetic impingement, dislocation, and polyethylene wear [28, 29]. Marchetti *et al.* [28] examined frequency and risk factors of prosthetic impingement in THA and reported that impingement was found in 51.4% and was severe in 31.3%. Shon *et al*. [29] also pointed out that more than half retrieved acetabular components showed impingement. Hara *et al*. [12] reported that liner-to-neck contact was observed in 36% of hips without component malpositioning during golf swing.

Hula is the traditional dance of Native Hawaiians, performed by men and women of all ages, and associated with improved physical function, lowering of systemic blood pressure, prevention of cardiac problems, reduced psychological stress, and improved self-regulatory ability [30–32]. Kaholokula *et al*. reported that movements can vary in intensity and duration, depending on the choreography of the dance, tempo of music, and skill level of the dancer, and can be modified for people with limited physical capacity [30, 31]. In terms of hula dance, both kao and kaholo demonstrated small hip flexion/extension, adduction/abduction, and internal/external rotation. No excessive hip movement or liner-to-neck contact was found during either kao or kaholo. No previous report is available of the *in vivo* three-dimensional kinematics of hula dance even in normal joints. The present study showed that orthopedic surgeons could encourage patients to participate in hula dance and enjoy their active lifestyles after THA.

In our report of two cases, we only evaluated the kinematics of the hip joint during two specific postures in classical ballet and hula dance. Different individuals would have significant differences between dancing styles, the spine and pelvic mobility, expertise, mood, and so on [10, 33, 34]. Therefore, this study did not clearly demonstrate which styles of dancing could be recommended or may be unsafe in other patients. Further studies with larger cohorts and a control group are desirable to deepen our understanding of the substantial inter-patient variability especially for the personal style of the dancer and skill levels [10, 33, 34]. Although kinematic processing of radiographic measurements is still challenging, time intensive, and requires the risk of radiation exposure, it does represent an important data-driven approach to provide feedback on sports-specific advice for each patient.

## Conclusion

We demonstrated visualization of hip motion during recreational dance activities performed after THA and quantification of the *in vivo* dynamic kinematics. No excessive, but gradual movements of hip flexion/extension, adduction/abduction, and internal/external rotation were demonstrated during both classical ballet and hula dance. Therefore, dance activities are recommended after THA in these cases. Kinematic data under weight-bearing conditions could be beneficial for advising patients who enjoy their active lifestyles, according to the type of physical activity.

## Abbreviations

CAD: Computer-aided design
CT: Computed tomography
DRRs: Digitally reconstructed radiographs
HHS: Harris Hip Score
OA: Osteoarthritis
OHS: Oxford Hip Score
ROM: Range of motion
TEA: Transe-epicondylar axis
THA: Total hip arthroplasty
UCLA: University of California-Los Angeles
